# Efficacy of Low-Dose Intravenous Immunoglobulin Combined With Immunosuppression Modification in the Treatment of BK Polyomavirus Nephropathy: A Case Series

**DOI:** 10.7759/cureus.99218

**Published:** 2025-12-14

**Authors:** Mahesh Eswarappa, Jaish George, Gurudev KC, Rajshekar R, Gireesh M S, Pooja Prakash Prabhu, Hamsa Reddy, Mohammad Yousuff, Rakesh Veluru

**Affiliations:** 1 Nephrology, Ramaiah Medical College, Bangalore, IND

**Keywords:** bk virus nephropathy (bkvn), intravenous immunoglobulin (ivig), renal function, renal transplant recipient, systemic tacrolimus

## Abstract

New and powerful immunosuppression agents presently available for maintenance immunosuppression and for the treatment of acute and chronic rejection in renal transplantation have led to the emergence of opportunistic infections. One such infection is BK virus nephropathy, leading to graft dysfunction and graft loss. Apart from the reduction and manipulation of immunosuppressive medications, various treatment options such as intravenous immunoglobulin (IVIg), cidofovir, leflunomide, and fluoroquinolones have been tried to treat BK virus nephropathy. We report our experience with low-dose IVIg in six renal transplant recipients with BK polyomavirus nephropathy. We suggest that low-dose IVIg can be a promising treatment option to treat and prevent a decline in renal function and subsequent graft loss.

## Introduction

Modern immunosuppressive regimens have substantially reduced rejection rates and enhanced transplant outcomes. Nevertheless, these powerful medications increase vulnerability to opportunistic infections and malignancies, potentially compromising graft longevity [1]. BK polyomavirus reactivation presents a particularly challenging complication, as this opportunistic pathogen can lead to transplant failure [2]. Latent BK polyomavirus becomes pathogenic under immunosuppressed conditions, triggering tubulointerstitial inflammation in up to 8% of patients [3].

Currently, no universally accepted therapeutic protocol exists for BK polyomavirus infection. Decreasing immunosuppressive therapy remains the primary intervention strategy [4]. Various adjunctive treatments, including leflunomide, cidofovir, intravenous immunoglobulin (IVIg), and fluoroquinolones, have undergone limited clinical evaluation with inconsistent results [5-7]. IVIg possesses immunomodulatory capabilities that extend beyond simple antibody supplementation. In transplant medicine, its applications span multiple domains, including treatment of antibody-mediated rejection, desensitization of highly sensitized transplant recipients, and treatment of post-transplant infectious complications like parvovirus B19, cytomegalovirus, and BK virus nephropathy (BKVN) [8,9].

We present our institutional experience with a combination therapy protocol for managing BKVN, consisting of low-dose IVIg combined with strategic immunosuppression modification. This integrated therapeutic approach, involving concurrent switch from tacrolimus to cyclosporine, discontinuation of mycophenolate mofetil, initiation of leflunomide, and administration of low-dose IVIG, represents a potentially cost-effective strategy that warrants investigation, particularly given resource constraints prevalent in many healthcare settings. We acknowledge that the simultaneous nature of these interventions precludes the determination of the independent contribution of each therapeutic component.

## Materials and methods

This was a retrospective case series conducted at Ramaiah Medical College and Hospital, Bangalore, India. The study was approved by the Ethics Committee of Ramaiah Medical College (approval number: MSRMC/EC/STU-DM1633/2025).

Study population

Inclusion criteria were adult (age >18 years) renal transplant recipients with biopsy-proven BKVN, elevated serum creatinine at presentation, and BK viremia confirmed by quantitative PCR in plasma. Patients with concurrent acute rejection, other active viral infections (cytomegalovirus, Epstein-Barr virus), a history of hypersensitivity to IVIg, and incomplete follow-up data were excluded. There were six cases of biopsy-proven BKVN that fulfilled the inclusion/exclusion criteria in the last eight years in our unit.

Data collection

Case files were reviewed, and demographic profiles and immunosuppression medications were noted. Each patient demonstrated elevated serum creatinine levels upon initial assessment with histologically confirmed BKVN. Their biopsy reports were noted. The entire cohort received IVIg at a dosage of 100 mg/kg administered daily for five days, with subsequent weekly doses continued for six weeks along with modification in their immunosuppression therapy. The improvement in graft function and decrease in viral load were noted, and patients were further followed up. The graft and patient survival were noted to date.

Diagnostic criteria for BKVN

BKVN was diagnosed based on: (i) histological evidence on renal allograft biopsy showing characteristic viral cytopathic changes with positive immunohistochemical staining for simian virus 40 (SV40) large T antigen, graded according to the Working Proposal classification system [10] (Class A: minimal tubular involvement, Class B: moderate tubular involvement with focal tubular atrophy/interstitial fibrosis, Class C: severe involvement with extensive fibrosis), (ii) presence of elevated plasma BK viral load measured by quantitative real-time polymerase chain reaction (qRT-PCR), and (ii) exclusion of other causes of allograft dysfunction including acute cellular rejection, antibody-mediated rejection, calcineurin inhibitor toxicity, and other viral infections.

Monitoring protocols

BK viral load monitoring was performed using qRT-PCR on plasma samples with a lower limit of detection of 250 copies/mL (analytical range: 250-10,000,000 copies/mL). Viral load testing was conducted at baseline (pre-treatment), at two months post-treatment initiation, and then at three-month intervals (six months, nine months, 12 months) for the first year.

Renal function was monitored through serum creatinine measurements obtained at baseline, weekly during the first month of treatment, then monthly for six months, and subsequently every three months. Tacrolimus trough levels were measured by liquid chromatography-tandem mass spectrometry (LC-MS/MS) with a target range of 5-8 ng/mL prior to BKVN diagnosis, and cyclosporine C0 trough levels were maintained at 100-150 ng/mL after the switch. Clinical monitoring for signs of acute rejection included surveillance of serum creatinine elevations (>0.3 mg/dL increase from baseline), decreased urine output, and allograft tenderness, with protocol or for-cause biopsies performed as clinically indicated.

IVIg product information

All patients received commercially available intravenous immunoglobulin preparations manufactured in India and pooled from local donor populations.

Critical Limitation

Anti-BK virus neutralizing antibody titers were not measured or standardized in any of the administered IVIg batches. Commercial IVIg preparations in India are not routinely characterized for anti-BK neutralizing antibody content, and batch-to-batch variability in anti-BK antibody titers is expected, given that donor pools reflect local BK seroprevalence patterns. The absence of titer quantification represents a significant limitation in establishing the biological mechanism underlying the observed clinical responses.

Immunological monitoring

Systematic measurement of donor-specific antibodies (DSA) using solid-phase assays (Luminex single-antigen bead technology) was not routinely performed before or after treatment in this retrospective case series. HLA antibody screening data were not available for analysis. Acute cellular or antibody-mediated rejection was assessed clinically through monitoring of serum creatinine and histologically when biopsies were performed for cause.

## Results

There were six patients with biopsy-proven BKVN identified in our institute for the study. Of these, four patients underwent renal transplantation from a live-related ABO-compatible donor. One patient received a kidney from an unrelated living donor, while another received a deceased donor transplant. Every patient in the series had received two doses of Injection Basiliximab as induction therapy on day 0 and day 4. The postoperative period was uneventful in all except the fourth patient, who developed sudden oliguria on the fifth postoperative day. This patient underwent graft biopsy, which showed antibody-cellular rejection class 2b, which was managed by injection of anti-thymocyte globulin (ATG) 1.5 mg/kg, and he recovered with baseline creatinine 1.12 mg/dL. All patients presented with graft dysfunction. One patient presented with severe graft dysfunction requiring hemodialysis. All of these patients were on routine monthly follow-up, and their serum creatinine was periodically monitored. The characteristics are given in Table 1.

**Table 1:** Clinical characteristics and outcomes of patients with BK virus nephropathy treated with IVIg (N=6) BKVN: BK virus nephropathy; IVIg: intravenous immunoglobulin

Parameter	Patient Values	Reference Ranges
Age (years), mean ± SD	42.5 ± 8.3	-
Male gender, n (%)	4 (66.7%)	-
Donor type, n (%)		-
Live-related	4 (66.7%)	-
Live-unrelated	1 (16.7%)	-
Deceased donor	1 (16.7%)	-
Time to BKVN diagnosis post-transplant, n (%)		-
<6 months	3 (50%)	-
>1 year	3 (50%)	-
Baseline creatinine (mg/dL), mean	3.21	0.7-1.3 mg/dL
BK viral load (copies/mL), mean	92,993	<1,000 copies/mL
Tacrolimus level (ng/mL) at presentation, range	5-8	5-15 ng/mL
BKVN stage on biopsy, mean	Stage B (100%)	-
Post-treatment creatinine (mg/dL), mean	1.5	0.7-1.3 mg/dL
BK viral clearance at 2 months, n (%)	6 (100%)	-
Graft survival, n (%)	6 (100%)	-

**Figure 1:**
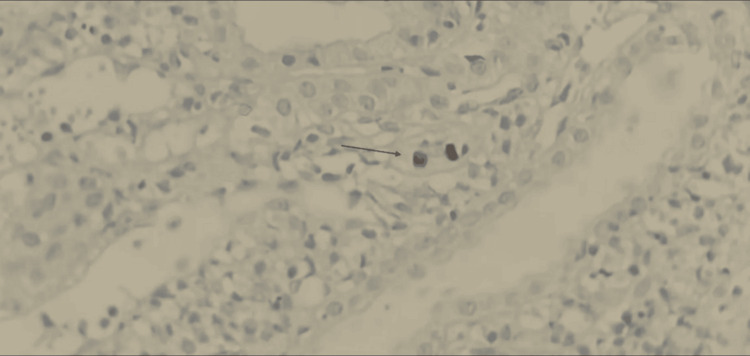
Immunohistochemistry confirmed the presence of simian virus 40 (SV40)-positive nuclei within tubular epithelial cells, consistent with BK virus nephropathy Renal cortical tissue displaying nuclear positivity for SV40 antigen within tubular epithelial cells (indicated by arrow), confirming BK polyomavirus infection. The positively stained nuclei appear dark brown, corresponding to viral inclusion bodies.  SV40 immunohistochemistry, original magnification ×400.

**Figure 2:**
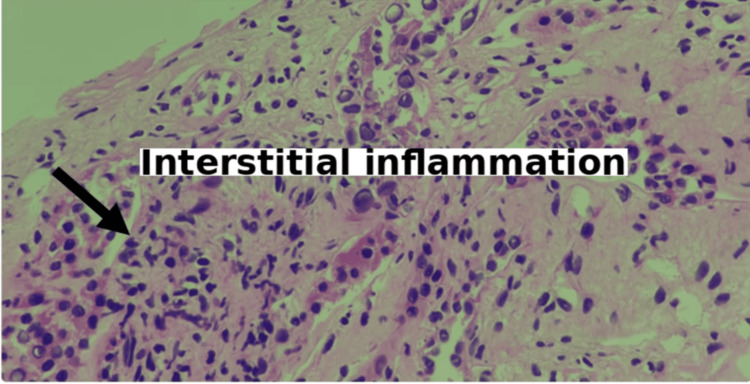
Renal biopsy demonstrating viral cytopathic changes consistent with Stage B BK virus nephropathy Renal cortical tissue exhibiting tubulointerstitial inflammation with enlarged tubular epithelial cells containing intranuclear viral inclusion bodies (arrow). The surrounding interstitium displays edema, lymphocytic infiltration, and tubular injury, consistent with Stage B BK virus nephropathy. Hematoxylin and eosin stain, original magnification ×400.

All six transplant recipients had tacrolimus levels maintained between 5 and 8 ng/mL measured by LC-MS/MS at presentation. Among the six patients, three patients had graft dysfunction after six months of transplant, and the other three patients had it after one year of transplant. The average baseline serum creatinine was 3.21 mg/dL at the time of presentation. Renal biopsy was performed in all the patients, which showed diffuse tubulointerstitial inflammation with simian virus 40-positive/viral inclusion bodies, which is graded as BK virus nephropathy stage B (Figures 1, 2).

All patients received combination therapy consisting of simultaneous immunosuppression modification and IVIg administration. The immunosuppressive regimen was modified as follows: tacrolimus was switched to cyclosporine (C0 trough levels 100-150 ng/mL), mycophenolate mofetil was discontinued, leflunomide was initiated (loading dose 100 mg/day for three days followed by maintenance dose 20 mg/day), and prednisolone 5 mg was continued. Concurrently, all patients received low-dose IVIg at 100 mg/kg daily for five days, followed by weekly doses continued for six weeks. Both cyclosporine and leflunomide possess documented independent anti-BK viral activity, making it impossible to isolate the specific contribution of IVIg to the observed outcomes.

Immunological safety and rejection monitoring

Clinical and histological monitoring for acute rejection was performed throughout the follow-up period. One patient (Patient 4) had experienced antibody-cellular rejection (Banff Class 2b) five days post transplant prior to BKVN diagnosis, which was successfully treated with anti-thymocyte globulin. Following initiation of combination therapy for BKVN (including immunosuppression reduction), none of the six patients developed clinical evidence of acute rejection based on serum creatinine monitoring during the treatment period or subsequent follow-up. No patient required a repeat allograft biopsy for suspected rejection after BKVN treatment was initiated. However, systematic donor-specific antibody (DSA) monitoring was not performed, precluding assessment of de novo alloantibody formation or subclinical antibody-mediated rejection.

Five patients (Patients 1, 2, 3, 5, and 6) achieved sustained viral clearance with stable graft function and were maintained on cyclosporine, leflunomide, and prednisolone. Patient 4 showed initial viral suppression but subsequently developed progressive graft dysfunction with persistent low-level viremia (250 copies/mL at 12 months) and elevated serum creatinine (9.6 mg/dL at 12 months), ultimately requiring return to maintenance hemodialysis. Therefore, the combination therapy protocol achieved complete viral clearance with preserved graft function in five of six patients (Table 2).

**Table 2:** Role of intravenous immunoglobulin for BK virus nephropathy in renal transplant recipients BKVN: BK virus nephropathy; IVIg: intravenous immunoglobulin

Transplant	Age/Sex	Date of Transplant	Date of Diagnosis (BKVN)	Basic disease	Induction	Biopsy	BK Copies	Baseline creatinine	Serum creatinine at presentation	Treatment	1^st^ month creatinine	3^rd^ month creatinine	12^th^ month Serum creatinine	Post-treatment serum creatinine (Post 1 year)	Post BK Copies
Deceased Donor Renal Transplant	40 years, male	November 2012	May 2013	Chronic Glomerulo Nephritis	Basiliximab	BKVN Stage B	68250 Copies /ml	1.1	3.94	IVIg 100 mg/kg For 5 days Once a week for 6 weeks leflunomide cyclosporine	4	3.11	2.96	1.5	NIL
Live Related ABO-Compatible Donor Maternal Aunt	18 years Female	August 2013	July 2014	Reflux Nephropathy	Basiliximab	BKVN Stage B	103478 Copies /ml	1.0	2.94	IIVIg 100 mg/kg For 5 days Once a week for 6 weeks leflunomide cyclosporine	2.91	1.86	1.68	1.5	NIL
Live Unrelated Renal Transplant	15 years Female	March 2015	March 2016	Primary Membranous Nephropathy	Basiliximab	BKVN Stage B	89476 Copies /ml	0.9	1.8	IVIg 100 mg/kg For 5 days Once a week for 6 weeks leflunomide cyclosporine	1.8	1.5	1.3	1.1	NIL
Live Related ABO-Compatible Renal Transplant Donor – Sister in Law	50 years Male	Jan 2018	Feb 2019	Diabetic Nephropathy	Basiliximab	BKVN Stage B	320000 Copies/ml	0.9	3.37	IVIg 100 mg/kg For 5 days Once a week for 6 weeks leflunomide cyclosporine	4	5.2	5.8	9.6	250 Copies/ml
Live Related Renal Transplant Donor - Wife	54 years Female	January 2020	October 2021	Diabetic Nephropathy	Basiliximab	BKVN Stage B	132000 copies/ml	1.1	3.08	IVIg 100 mg/kg For 5 days Once a week for 6 weeks leflunomide cyclosporine	3	2.86	2.01	1.5	100 Copies/ml
Live Related ABO-Compatible Donor Husband	53 years Female	May 2021	Nov 2021	Diabetic Nephropathy	Basiliximab	BKVN Stage B	71,760 Copies /ml	1.3	5.3	IVIg 100 mg/kg For 5 days Once a week for 6 weeks leflunomide cyclosporine	5.4	4.6	3.4	2.1

## Discussion

BKVN represents one of the most complex post-transplant infectious challenges requiring careful management. Multiple factors predispose transplant recipients to BKVN development. Potent immunosuppression, particularly lymphocyte-depleting induction agents and high-dose corticosteroids administered for rejection treatment, creates conditions favoring viral reactivation [11-16]. Additional contributors include initial graft ischemia-reperfusion injury, human leukocyte antigen (HLA) incompatibility between donor and recipient, and the magnitude of BK viremia itself. Immunosuppression reduction constitutes the most common therapeutic intervention for BKVN in renal transplant recipients; however, this approach does not uniformly guarantee renal function improvement.

In recent years, IVIg has been increasingly utilized in BKVN management [17-21]. IVIg provides passive immunity against the BK virus through donor-derived antibodies present in pooled immunoglobulin preparations. The therapeutic mechanism operates via virus-specific neutralization, preventing viral entry into host cells. Emerging evidence supports combining IVIg therapy with immunosuppression reduction, as this dual strategy addresses both viral replication and immune system reconstitution.

Jamboti (2016) reported two key mechanistic findings relevant to BKVN management: cyclosporine exhibited direct anti-replicative effects against BK virus in renal tubular and bladder epithelial cells, while commercial IVIG preparations demonstrated broad neutralizing activity across multiple BK viral genotypes [18]. These observations provide a biological rationale for therapeutic strategies combining calcineurin inhibitor modification with IVIg administration.

Anti-BK neutralizing antibody titers and geographic considerations

A critical factor influencing IVIg efficacy in BKVN is the anti-BK virus neutralizing antibody titer in the administered preparation. IVIg's therapeutic mechanism in viral infections depends on the passive transfer of virus-specific neutralizing antibodies present in pooled immunoglobulin from healthy donors. Jamboti demonstrated that commercial IVIg preparations exhibit broad neutralizing activity across multiple BK viral genotypes, suggesting adequate antibody representation in donor pools [18]. However, anti-BK antibody titers vary substantially between different IVIg products and batches, depending on the seroprevalence of BK virus in the donor population and the number of donors contributing to each pool.

In the Indian context, commercial IVIg products are manufactured from plasma pooled from local donor populations. BK virus seroprevalence in healthy Indian adults has been reported at approximately 60-90% in limited studies [22], suggesting that Indian IVIg pools likely contain anti-BK antibodies. However, neither manufacturers nor regulatory authorities mandate quantification or standardization of anti-BK neutralizing antibody titers in IVIG products. Therefore, the actual neutralizing potency of the IVIg batches administered in our study remains unknown, introducing uncertainty regarding the biological mechanism underlying our observed outcomes.

The reported efficacy of our low-dose protocol (100 mg/kg) compared to standard high-dose regimens (1-2 g/kg) raises two possible interpretations: (i) the Indian IVIg batches used may have contained exceptionally high anti-BK neutralizing antibody titers, enabling efficacy at lower doses; or (ii) the primary therapeutic benefit derived from the concurrent immunosuppression modifications (cyclosporine switch and leflunomide addition) rather than IVIg-mediated passive immunity. Without titer quantification, we cannot distinguish between these possibilities. This limitation is particularly important when considering the generalizability of our low-dose regimen to other settings where IVIg products may differ in anti-BK antibody content.

Our institutional experience with low-dose IVIg demonstrated substantial reductions in BK polyomavirus plasma levels, with complete viral clearance achieved in all cases, accompanied by improved renal function. These outcomes compare favorably with published reports utilizing significantly higher IVIg doses, suggesting a potential therapeutic window at lower dosing that warrants further investigation.

Combination therapy methodological considerations

A critical methodological consideration in interpreting our results is the simultaneous implementation of multiple therapeutic interventions. Our protocol involved four concurrent modifications: (i) calcineurin inhibitor switch (tacrolimus to cyclosporine), (ii) cessation of mycophenolate mofetil, (iii) addition of leflunomide, and (iv) IVIg administration. Both cyclosporine and leflunomide have established independent antiviral properties against the BK virus. Jamboti demonstrated that cyclosporine exhibits direct anti-replicative effects against the BK virus in renal tubular and bladder epithelial cells [18]. Similarly, leflunomide's active metabolite, teriflunomide, inhibits de novo pyrimidine synthesis, thereby suppressing viral replication [5]. The simultaneous implementation of these interventions represents a pragmatic clinical approach but precludes the determination of the independent contribution of each component, including IVIg, to the observed viral clearance and functional improvement. Therefore, our findings should be interpreted as demonstrating the efficacy of this combination therapeutic strategy rather than IVIg monotherapy.

Previous investigations support IVIg's efficacy in BKVN management. Sener et al. (2006) reported favorable long-term outcomes using high-dose IVIg (2 g/kg) alongside immunosuppression modification, with graft preservation extending beyond one year in the majority of cases [17]. Notably, their higher dosing regimen contrasts with our low-dose protocol, yet both approaches achieved viral clearance and functional improvement.

Sharma and colleagues reported IVIg as successful rescue therapy in a pediatric case where cidofovir proved inadequate [7]. Their multi-dose IVIg protocol achieved substantial viral load reduction and sustained renal function stabilization over six months, supporting IVIg's role in managing refractory BKVN cases.

Vu et al. (2015) demonstrated IVIg's utility as salvage therapy for patients unresponsive to standard anti-polyomavirus treatments [19]. Among recipients receiving IVIg after first-line therapies failed, the majority achieved viral clearance with excellent graft and patient survival at one year. These results support IVIg's safety and efficacy as rescue therapy, particularly when combined with leflunomide and immunosuppression modification. Shah et al. evaluated higher-dose IVIg (1.0 g/kg) combined with leflunomide, demonstrating rapid viral load reduction within one month [20]. Follow-up biopsies frequently revealed concurrent resolution of both rejection and BKVN, suggesting potential dual benefits of this therapeutic combination.

Our combination protocol included substantially lower IVIg doses (100 mg/kg)-approximately one-tenth of previously reported IVIg monotherapy regimens and yet achieved favorable outcomes. This finding aligns with reports by Matsumura et al. [21] and Anyaegbu and Hmiel [23], who similarly documented success with reduced-dose therapy. However, interpretation of these low-dose successes must be tempered by several considerations. First, without quantification of anti-BK neutralizing antibody titers in the administered IVIg preparations, we cannot verify whether adequate passive immunity was achieved at this low dose or whether the observed viral clearance primarily resulted from concurrent immunosuppression modifications. Second, if Indian IVIg batches contain higher anti-BK antibody titers than products used in other geographic regions (due to differences in donor population BK seroprevalence), our low-dose regimen may not be directly generalizable to other settings. Third, the concurrent use of agents with independent antiviral activity (cyclosporine and leflunomide) may have reduced the necessary IVIG dose threshold for efficacy. These uncertainties highlight the need for future studies that include IVIG titer quantification and separate treatment arms to establish optimal dosing strategies.

Immunological safety during immunosuppression reduction

A critical consideration when reducing immunosuppression to manage BKVN is the risk of precipitating acute rejection or inducing de novo DSA formation. Bartel et al. (2011) demonstrated that immunosuppression reduction for viral infections increases the risk of both cellular and antibody-mediated rejection [13]. In this context, IVIg serves dual roles: providing anti-BK neutralizing antibodies for viral control while potentially offering immunomodulatory effects that mitigate rejection risk during reduced immunosuppression. Jordan et al. (2011) reviewed IVIg's immunomodulatory mechanisms in transplantation, including Fc receptor blockade, complement inhibition, and modulation of T-cell and B-cell responses, all of which theoretically reduce alloimmunization risk [8].

However, we acknowledge a significant limitation in our study: the absence of systematic DSA monitoring. None of our patients developed clinical evidence of acute rejection (based on serum creatinine surveillance) during or after BKVN treatment, suggesting adequate immunosuppression was maintained despite the therapeutic modifications. Nevertheless, subclinical antibody-mediated rejection or de novo DSA formation cannot be excluded without systematic Luminex single-antigen bead testing. The question of whether our low-dose IVIg protocol (100 mg/kg) provided sufficient immunomodulation to prevent alloimmunization during the period of reduced net immunosuppression remains unanswered. Standard high-dose IVIg regimens (2 g/kg) used for desensitization and rejection treatment provide robust immunomodulation, but whether low-dose IVIg offers comparable protective effects against de novo DSA formation is uncertain.

Furthermore, the immunosuppression modifications themselves may have influenced alloimmunization risk. The switch from tacrolimus to cyclosporine represents a lateral move within the calcineurin inhibitor class, maintaining T-cell suppression. The substitution of leflunomide for mycophenolate mofetil may actually provide comparable or superior prevention of alloimmunization, as leflunomide's active metabolite inhibits both T-cell and B-cell proliferation. Therefore, the net immunosuppression level may have been less dramatically reduced than suggested by the qualitative description of the regimen changes. These considerations highlight the need for future prospective studies incorporating systematic DSA monitoring before and after BKVN treatment to establish the immunological safety profile of low-dose IVIG protocols.

Recent comparative investigations by Rasaei et al. (2023) demonstrated enhanced viral load reduction with combination therapy versus IVIg monotherapy [24]. Their findings suggest that adding leflunomide to IVIg treatment produces superior viral suppression over three months, a pattern consistent with our case series, where combination immunosuppression modification and IVIg yielded favorable outcomes.

Limitations

This study has several critical limitations that affect the interpretation of our findings.

Combination Therapy Confounding

The most significant limitation is our inability to determine the independent contribution of IVIg versus immunosuppression modification (specifically, the switch to cyclosporine and addition of leflunomide, both of which have documented anti-BK viral properties). The simultaneous implementation of all therapeutic components represents standard clinical practice in managing BKVN but creates methodological confounding that prevents isolation of IVIg's specific effect. Our study design does not permit differentiation between the efficacy attributable to low-dose IVIg versus the antiviral effects of cyclosporine and leflunomide. Future randomized controlled trials with separate treatment arms (IVIg plus immunosuppression modification versus immunosuppression modification alone) would be required to establish IVIg's independent therapeutic contribution.

Lack of Anti-BK Neutralizing Antibody Titer Quantification

We did not measure anti-BK virus neutralizing antibody titers in the IVIg batches administered to our patients. IVIg's proposed therapeutic mechanism in BKVN relies on passive transfer of BK-specific neutralizing antibodies, yet commercial IVIg products in India are not standardized for anti-BK antibody content. Batch-to-batch variability in neutralizing titers is expected based on donor pool composition and BK seroprevalence in the source population. Without titer quantification, we cannot establish whether the observed clinical responses resulted from adequate passive immunity via IVIg, from the concurrent immunosuppression modifications with independent antiviral activity, or from synergistic effects of both. The biological plausibility of achieving complete viral clearance with low-dose IVIg (100 mg/kg, one-tenth of standard regimens) remains speculative without verification of adequate neutralizing antibody delivery. Future investigations should include pre-administration measurement of anti-BK neutralizing antibody titers in IVIg products and assessment of recipient anti-BK antibody levels before and after IVIg administration to establish pharmacodynamic efficacy.

Absence of Donor-Specific Antibody Monitoring

We did not perform systematic measurement of DSA before or after treatment using Luminex single-antigen bead technology. This represents a critical gap in assessing immunological safety during the period of reduced immunosuppression. While none of our patients developed clinical evidence of acute rejection, we cannot exclude subclinical antibody-mediated rejection or de novo DSA formation. A key theoretical benefit of IVIg in the context of immunosuppression reduction is its immunomodulatory properties that may prevent alloimmunization. However, whether our low-dose IVIg regimen (100 mg/kg) provided adequate immunomodulation to prevent de novo DSA formation remains unknown. Standard high-dose IVIg protocols (2 g/kg) used for desensitization provide robust immunosuppression, but the immunomodulatory threshold of low-dose IVIg is not established. Future studies must incorporate systematic DSA monitoring (pre-treatment baseline, during treatment, and long-term follow-up) to validate the immunological safety of low-dose IVIg protocols and to determine whether they offer sufficient protection against alloimmunization during therapeutic immunosuppression reduction for BKVN management.

Additional Limitations

The small sample size (n=6) limits the generalizability of our findings and prevents statistical analysis of treatment efficacy. This was a retrospective case series without a control group, making it difficult to determine treatment effectiveness compared to alternative approaches. The lack of standardized dosing protocols and follow-up periods may have introduced variability in treatment responses. The study was conducted at a single center, which may not reflect diverse patient populations or practice patterns at other institutions. Long-term follow-up data beyond the immediate post-treatment period were limited, and the durability of treatment response remains uncertain. We did not assess the potential adverse effects of IVIg therapy in detail. Finally, the cost-effectiveness of low-dose IVIg compared to other treatment strategies was not evaluated. Future prospective, multicenter, randomized controlled trials with larger sample sizes are needed to validate our findings and establish optimal treatment protocols for BKVN.

## Conclusions

Combination therapy consisting of low-dose IVIg, calcineurin inhibitor switch to cyclosporine, and addition of leflunomide represents a promising therapeutic strategy for managing BK viremia and BKVN. Our case series demonstrates that this integrated approach achieved viral clearance in all patients and preserved graft function in all patients. However, the simultaneous implementation of multiple interventions with independent anti-BK viral properties prevents the determination of IVIg's specific contribution to these outcomes. Three critical methodological limitations affect interpretation: (i) the inability to isolate IVIg's effect from concurrent immunosuppression modifications with documented antiviral activity, (ii) the absence of anti-BK neutralizing antibody titer quantification in the administered IVIg preparations, and (iii) the lack of systematic donor-specific antibody monitoring to assess immunological safety during immunosuppression reduction.

Future randomized controlled trials comparing IVIg plus immunosuppression modification versus immunosuppression modification alone are necessary to establish whether low-dose IVIg provides additional benefit beyond the antiviral effects of cyclosporine and leflunomide. Such studies should incorporate measurement of anti-BK neutralizing antibody titers in IVIg products and recipient serum to establish pharmacodynamic relationships between IVIg dose, antibody delivery, and clinical outcomes. Critically, future investigations must incorporate systematic donor-specific antibody monitoring to establish the immunological safety profile of low-dose IVIg protocols, specifically whether they provide adequate immunomodulation to prevent de novo alloimmunization during the period of reduced net immunosuppression required for BKVN management. Despite these limitations, our experience suggests that combination therapy with low-dose IVIg represents a potentially cost-effective approach that warrants further investigation, particularly in resource-limited settings. Viral clearance rate and preserved graft function in all patients demonstrate the clinical promise of this strategy, although additional research is needed to optimize protocols and establish long-term efficacy and safety.
